# Operative and Survival Outcomes of Robotic-Assisted Surgery for Colorectal Cancer in Elderly and Very Elderly Patients: A Study in a Tertiary Hospital in South Korea

**DOI:** 10.1155/2022/7043380

**Published:** 2022-01-30

**Authors:** Hugo Cuellar-Gomez, Siti Mayuha Rusli, María Esther Ocharan-Hernández, Tae-Hoon Lee, Guglielmo Niccolò Piozzi, Seon-Hahn Kim, Cruz Vargas-De-León

**Affiliations:** ^1^Division of Colorectal Surgery, Department of Surgery, Korea University Anam Hospital, Korea University College of Medicine, Seoul, Republic of Korea; ^2^Escuela Superior de Medicina, Instituto Politécnico Nacional, Plan de San Luis y Díaz Mirón SN, Col. Casco de Santo Tomás, Alcaldía Miguel Hidalgo, C. P. 11340, Ciudad de México, Mexico; ^3^División de Investigación, Hospital Juárez de México, Av Instituto Politécnico Nacional 5160, Alcaldía Gustavo A. Madero, CP. 07760, Ciudad de México, Mexico; ^4^Facultad de Matemáticas, Universidad Autónoma de Guerrero, Av. Lázaro Cárdenas S/N, Cd. Universitaria Sur, C.P. 39087, Chilpancingo, Guerrero, Mexico

## Abstract

**Materials and Methods:**

Data of all patients ≥75 years who underwent a robotic-assisted curative resection in Korea University Anam Hospital, Seoul, South Korea, between January 2007 and January 2021 were extracted from a prospectively maintained colorectal cancer database. Patients were subdivided into the three groups according to the age: youngest-old (YO: 75–80 years), middle-old (MO: 81–85), and oldest-old (OO: ≥86 years). Intraoperative findings, postoperative, and oncological outcomes were compared between the groups.

**Results:**

Seventy-six consecutive patients (female 52.6%) were included; mean age was 80 years (SD 0.33); mean body mass index (BMI), 23.8 20.9 kg/m^2^ (SD 3.58); mean total operative time, 279 min (SD 80.93); mean blood loss, 186 ml (SD 204.03); mean postoperative length of stay, 14 days (SD 12.03). Major complications were seen in 2.1% of patients. The 30-day mortality rate was 0%. Average number of lymph node harvested was 20.9 (SD 12.33). Postoperative complications were not statistically different between the groups. Mean follow-up time for cancer-specific survival (CSS) was 99.28 months for the YO, 72.11 months for MO, and 31.25 months for OO groups (*p* = 0.045). The CSS rates at 5 years were 27.0%, 21.0%, and 0%, respectively. Recurrence risk was 10.50 times higher in the OO group than the others (adjusted HR, 95% CI 1.868–59.047, *p* = 0.008). In the multivariable analysis, TNM stage was not a risk factor for CSS in all groups. The number of the harvested nodes was a protective factor for recurrence (HR of 0.932, 95% CI 0.875–0.992, *p* = 0.027) and CSS (HR of 0.928, 95% CI 0.861–0.999, *p* = 0.047) in elderly patients.

**Conclusion:**

Robotic surgery is highly feasible in elderly and very elderly colorectal cancer patients, providing a favorable operative safety profile and an acceptable cancer-specific survival outcome.

## 1. Introduction

Colorectal cancer (CRC) is a common and life-threatening disease worldwide [1], with Asia contributing the highest, 1, 009,400 (52.3%) of incident cases and 506, 499 (54.2%) of deaths in 2020 [2]. South Korea in 2018 had the second highest incidence rate of CRC in Asia, with currently positioning it at the fourth most common cause of cancer death [3, 4]. Many population data reported that approximately 30–40% of CRC cases occur in patients aged above 75 years [1, 5]. According to the World Health Organization, when an aging rate (the proportion of a society's population aged 65 or older) exceeds 7%, 14%, and 21%, a society is defined as “aging society,” “aged society,” or “super-aged society,” respectively [6]. In 2017, South Korea had officially become an aged society, with more than 14% of its citizens ≥65 years [7]. Aging is characterized by a rising susceptibility to develop multiple chronic diseases; therefore, it represents the major risk factor for multimorbidity. According to the International Society of Geriatric Oncology (SIOG), older patients with CRC undergoing surgery should receive the same treatment as their younger counterparts but with an adjustment of treatment strategy in case of comorbidity, limited physiologic reserves, and emergency situations. However, it has been demonstrated that favorable long-term outcomes can be achieved by surgery alone [8, 9], and age is not independently associated with complications after surgery for CRC [10]. Therefore, the surgical strategy should be focused on not increasing the morbidity and mortality rate in elderly patients. So far randomized control trials like ASCOSOG [11], ALaCaRT [12], COREAN, [13] MRC CLASICC [14], and COLOR II [15] have demonstrated that although laparoscopic surgery has similar long-term outcomes and morbidity/mortality compared to open surgery, the short-term outcomes were better after laparoscopic surgery [16]. Robotic-assisted surgery provides several advantages over the laparoscopic approach such as the 3D vision and the absence of tremors in the instruments, which could lead to possible benefits in oncological or short-term outcomes [17], and has been demonstrated to be a feasible and safe alternative approach to CRC surgical treatment [18]. Some studies suggest that robotic-assisted colorectal surgery (RACS) could potentially offer better short-term outcomes and reduction in conversion to open rates compared to laparoscopic surgery, especially when applied in selected patients [19–21]. Some studies have demonstrated the feasibility of robotic-assisted surgery in elderly patients with cancer [22, 23].

The aim of this study was to evaluate the outcomes of RACS in elderly and very elderly patients, focusing on demographic characteristics, surgical, oncological, and postoperative outcomes, overall survival (OS) rate, cancer-specific survival (CSS) rate, and cumulative recurrence rate (CRR) in a high-volume RACS tertiary center in South Korea.

## 2. Patients and Methods

### 2.1. Patients

A retrospective study was performed by evaluating 76 consecutive elderly patients submitted to robotic-assisted CRC surgery with the da Vinci® S, Si or Xi Surgical Systems (Intuitive Surgical Inc., Sunnyvale, CA, USA). The data were extracted from a prospectively maintained colorectal database with a total of 4,681 patients who underwent surgery for CRC from January 2007 to January 2021 at Korea University Anam Hospital, a tertiary referral center in South Korea. In the study period, RACS was performed in 947 patients.

The patients were divided into three groups according to their age: youngest-old (YO: 75–80 years), middle-old (MO: 81–85), and oldest-old (OO: ≥86 years) [24]. The exclusion criteria were as follows; patients with locally recurrent cancer, patients with indeterminate lesion(s) in the liver, and/or the lung at the time of surgery but proven as metastatic disease during postoperative follow-up.

Clinical staging was performed via colonoscopy with biopsy, thoracic-abdominal-pelvic computed tomography (CT), and abdominal-pelvic magnetic resonance imaging (MRI) for tumor staging. Pretreatment workup was carried out in all patients with cardiac and pulmonary risk evaluation. The indication for surgery in all cases was primary CRC. All cases were discussed in a multidisciplinary (MDT) meeting before treatment. The robotic approach was proposed to patients based on their general clinical conditions, tumor characteristics, and physician's preference. The final pathologic features were restaged according to the 8th edition of the American Joint Committee on Cancer (AJCC) staging system [25] at the time of data review.

### 2.2. Data Collection

Clinical data included the following: sex, age, body mass index (BMI), comorbidities, American Society of Anesthesiologists (ASA) score, and preoperative carcinoembryonic antigen (CEA). Surgical data included tumor location, type of procedure, operative time, conversion to open surgery, and estimated blood loss (EBL). Postoperative complications were reported according to the modified Clavien-Dindo classification (C-D) [26], together with the type of complication, length of hospital stay (LOS), and reoperation rate (patients reoperated within 30 days from initial surgery). Operative morbidity and mortality had been prospectively collected through a quality improvement meeting of our division, on a weekly basis, in the colorectal database since 2007. Tumor histological type, grading, and TNM stage were retrieved from the final pathological reports. Postoperative follow-up protocol included physical examination and serum CEA assay every three months for the first two years, thereafter every six months; chest and abdominopelvic CTs were taken every six months for the first two years then annually for the following years; colonoscopy and sigmoidoscopy were performed alternatively every six months for the first two years then colonoscopy annually in rectal cancer, but annual colonoscopy only in colon cancer. Additional tests, including pelvic MRI or positron emission tomography scan, were performed on an as-needed basis.

Recurrence was diagnosed through radiological detection of lesions with increasing size or by histological confirmation. Time to recurrence was defined as the interval between the date of initial surgery and the date of recurrence confirmation. Cumulative recurrence rate (CRR) was referred to as the cumulative probability of CRC recurrence occurring during follow-up. Overall survival (OS) was measured from the date of surgery to that of death or last follow-up. Cancer-specific survival (CSS) was measured from the date of surgery to cancer-related death. This study follows the STROBE statement for cohort studies [27]. The study was approved by the Institutional Review Board (2021AN0411) of Korea University Anam Hospital.

### 2.3. Statistical Analysis

Data are presented using mean (standard deviation (SD)) and counts (percentage) for numerical and categorical variables, respectively. According to the age of the patients, three groups were formed (YO, MO, and OO). Categorical variables were compared using Fisher's exact test, and continuous variables were compared using one-way analysis of variance (ANOVA) followed by post hoc analysis with the Tukey's test. OS, CSS, and CRR were estimated using the Kaplan–Meier method, and the curves were compared using the Breslow test. A multivariate Cox proportional hazard regression analysis was performed to identify variables independently associated with OS, CSS, and CRR. Hazard ratio (HR) was estimated as a measure of effect size of the variables included in the Cox regression. *p* values <0.05 were considered statistically significant. All analyses were performed with IBM SPSS Statistics for Windows, version 24 (IBM Corp., Armonk, NY, USA).

## 3. Results

### 3.1. Patient Demographics

The patients were subdivided according to their age in YO (*n* = 48), MO (*n* = 19), and OO (*n* = 9); female proportion was 62.5%, 26.3%, and 55.6%, for each group, respectively (*p* = 0.028). The lowest BMI (20.9 kg/m^2^ (SD 2.86), *p* = 0.030) and the highest CEA (10.30 UI (SD 11.39), *p* = 0.010) were observed in the OO group. Most patients were ASA II (YO 81.3%, MO 84.2%, and OO 66.7%, *p* = 0.645). Patient comorbidities showed heterogeneous tendency with cardiovascular and endocrine diseases were the most frequent in all groups: YO (47.8%, and 14.6%), MO (47.4% and 15.8%), and OO (33.3% and 22.3%), respectively (*p* = 0.128). The preoperative radiological stages II and III were the most frequent for all groups; YO 22% and 64.6%, MO 5.3% and 78.9%, and OO 0% and 6%, respectively, with no significant differences between them (*p* = 0.129). The neoadjuvant therapy was used for locally advanced cancers of the mid or distal rectum. Twenty-three patients (31.5%) received neoadjuvant therapy, long-course concurrent chemoradiotherapy (*n* = 19), short-course radiotherapy (*n* = 2), and chemotherapy (*n* = 2). Eighteen patients (23.6%) were treated with adjuvant chemotherapy based on FOLFOX or 5-fluorouracil (5-FU). One patient received palliative radiotherapy postoperatively. The clinical data are summarized in Table 1.

### 3.2. Surgical Data

The lower rectum (≤5 cm from the anal verge) was the most common tumor site in all groups (55.2%). Of those who had rectum resection, 68.0% required a stoma; 42 cases were diverted with temporary ileostomy, and seven had a permanent colostomy (abdominoperineal resection in 5 and Hartmann's procedure in 2). Mean total operative time was 279 min (SD 80.93), with a mean docking time of 10 min (SD 6.50). Mean EBL was 186 ml (SD 204.03). EBL showed significant difference between groups, with the YO group reporting the lowest (133.3 ml, SD 234.8) and the MO group the highest (290 ml, SD 17.88) (*p* = 0.009). No conversion from robotic to open surgery was reported.

A total of 69 patients (90.7%) had primary anastomosis and 63 (82.8%) had colorectal anastomosis. Only four patients (2.1%) developed anastomotic leakage with a risk of 0.3%. There was no 30-day operative mortality in our cohort.

Most patients (97.3%) did not require surgical management of complications (C-D ≤ II). Only two patients with C-D ≥ III (2.6%) were reoperated for anastomotic leakage, and both needed Hartmann's procedure. Mean postoperative hospital stay was 14.25 days (SD 12.03) with no statistical difference between groups (*p* = 0.579). Surgical data including the operative outcomes are summarized in Table 2.

### 3.3. Clinicopathological Characteristics and Oncological Outcomes

Mean resected lymph node number was 20.9 (SD 12.33), with the highest harvest in the OO (24.56 lymph nodes), no significant difference was found between groups (*p* = 0.648). The majority of patients had adenocarcinoma in the histology (96.0%). The (y)pT3 was more frequent in all groups (YO 64.6%, MO 78.9%, and OO 66.7%; *p* = 0.900). The (y)pN0 was the most frequent (YO 60.4%, MO 78.9%, and OO 44.4%; *p* = 0.184). Pathological TNM stage was similar between the groups (*p* = 0.127). Stage I and III were most frequent in YO (35.4% and 31.3%) and OO (22.2% and 55.6%), whereas MO presented stage II and III as most frequent with 47.4% and 36.3%, respectively. Stage IV had the same distribution in all groups. Pathologic data are summarized in Table 3.

### 3.4. Survival and Recurrence

**Figure 1:**
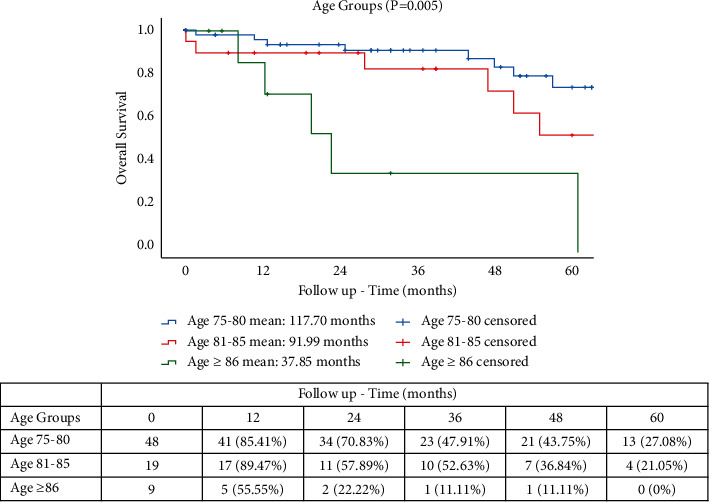
OS in elderly patients with RACS.

**Figure 2:**
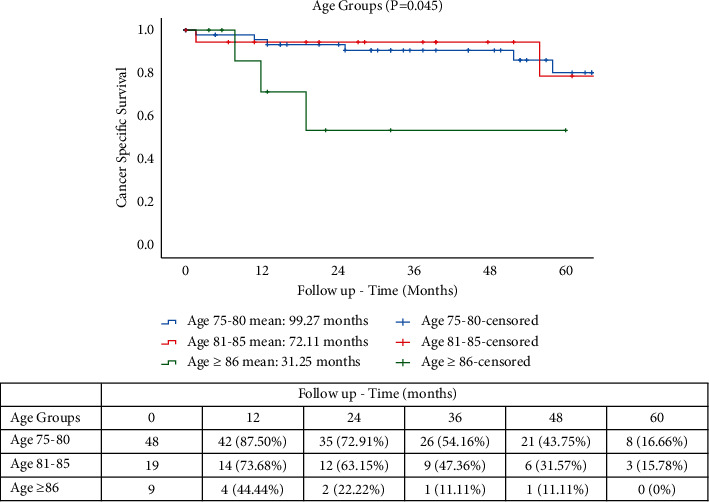
CSS in elderly patients with RACS.

**Figure 3:**
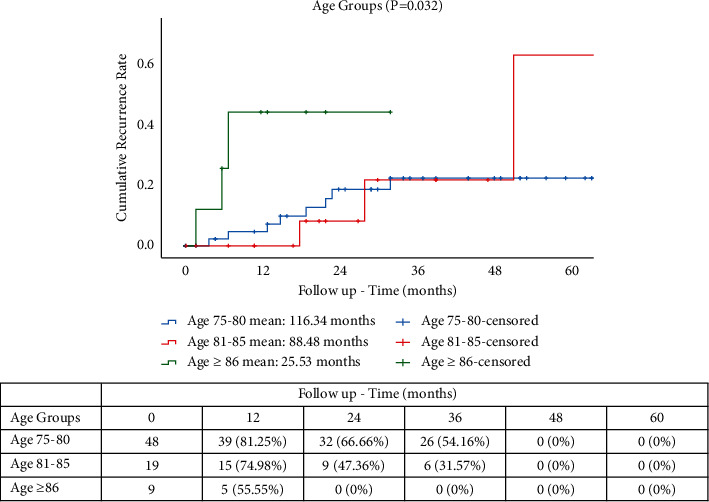
CRR in elderly patients with RACS.

**Figure 4:**
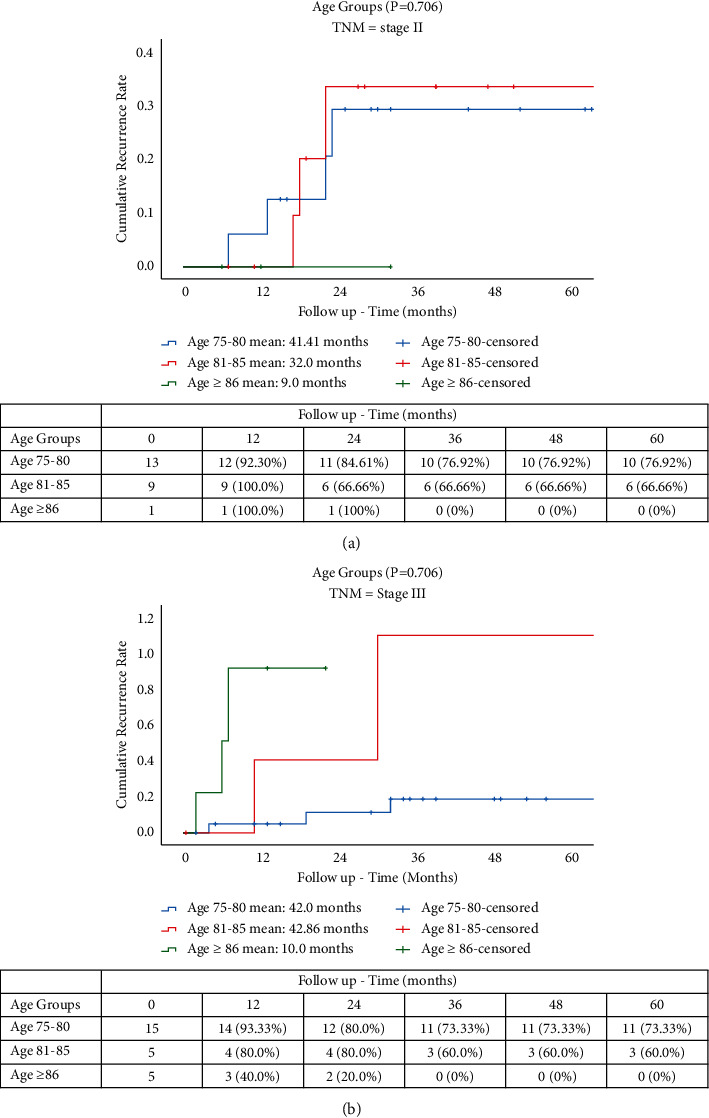
CRR in elderly patients with TNM. (a) Stage II. (b) Stage III.

**Figure 5:**
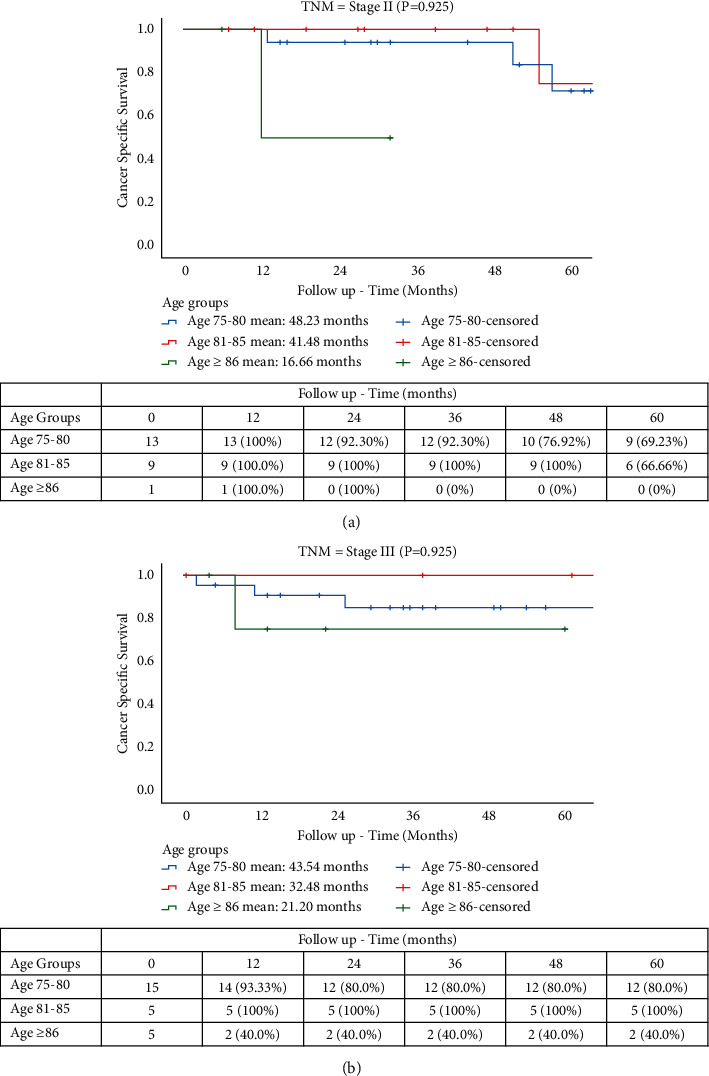
CSS in elderly patients with TNM. (a) Stage II. (b) Stage III.

Mean follow-up times for OS were 117.70 months for YO, 91.99 months for MO, and 37.85 months for OO groups (*p* = 0.045). OS rates were 16.66%, 3.0%, and 0%, respectively, for YO, MO, and OO (Figure 1). The CSS rates at 5 years were 16.66% (YO), 15.78% (MO), and 0% (OO), with mean follow-up times of 99.27, 72.11, and 31.25 months, respectively (*p* = 0.045) (Figure 2). The CRRs in patients with cancer-related death (Figure 3) at 5 years were 81.25%, 66.66%, and 54.16% at 12, 24, and 36 months for YO, 74.98% and 47.36% at 12 and 24 months for MO, and 55.55% for OO at 12 months with mean follow-up times of 116.34, 88.48, and 22.53, respectively (*p* = 0.032). A total of 15 patients (19.7%) developed a recurrence with 13 patients (17.1%) having a cancer-related death. A subgroup comparison between age and TNM stage II and III was carried out for CSS and CRR. The CRRs rates at 5 years were 76.92% (YO), 66.66% (MO), and 0% (OO) for TNM stage II (Figure 4(a)), with mean follow-up times of 41.41, 32.0, and 9.0 months, respectively (*p* = 0.706). The CRRs rates at 5 years were 73.33% (YO), 60.0% (MO), and 0% (OO) for TNM stage III (Figure 4(b)), with mean follow-up times of 42.0, 42.86, and 10.0 months, respectively (*p* = 0.706). The CSS rates at 5 years were 69.23% (YO), 66.66% (MO), and 0% (OO) for TNM stage II (Figure 5(a)), with mean follow-up times of 48.23, 41.84, and 16.66 months, respectively (*p* = 0.925), and CSS rates at 5-yeat were 80.0% (YO), 100% (MO), and 40.0% (OO) for TNM stage III (Figure 5(b)), with a mean follow-up time of 43.54, 32.48, and 21.10 months, respectively (*p* = 0.925).

### 3.5. Risk Factor Analysis for Prognosis

Results of adjusted multivariate Cox regression for OS, CSS, and recurrence are reported in Tables 4–6. In the multivariate analysis for OS; age (≥86 years) was a risk factor 25.553 times (adjusted HR, 95% CI 5.600–116.596, *p* = 0.001) compared to YO group, and cirrhosis was a risk factor 41.198 times compared to no comorbidities (adjusted HR, 95% CI 4.617–367.623, *p* = 0.001). The age (≥86 years) was a risk for recurrence 8.368 (adjusted HR, 95% CI: 1.547–45.248, *p* = 0.014) times higher compared to the YO group, and stages pN1 and pN2 had 4.341 (adjusted HR, 95% CI: 1.127–16.717, *p* = 0.033) and 6.669 (adjusted HR, 95% CI: 1.382–32.192, *p* = 0.018) times higher risk of tumor recurrence compared to stage N0 in all groups. Number of the harvested nodes was a protective factor for CSS (HR 0.928, 95% CI 0.861–0.999, *p* = 0.047) and against tumor recurrence (HR: 0.932, 95% CI: 0.875–0.992, *p* = 0.027). The pT stage and pathological TNM stage were not a risk factor for recurrence and CSS, respectively. Nevertheless, age ≥86 (OO) was a risk factor 9.087 times (adjusted HR, 95% CI 1.734–47.611, *p* = 0.009) compared to YO group for CSS.

## 4. Discussion

CRC is one of the most common malignancies in the world, especially in the elderly. Current trends in epidemiology indicate an acceleration of population aging, resulting in an increase in CRC incidence about 30 times greater risk than young people [28]. The management of CRC in elderly patients should be aggressive and similar to younger patients [8]. Age is not a contraindication for minimally-invasive surgery in CRC [29], and RACS has been proven to be feasible for this disease [30].

There is controversy in literature over the definition of elderly patients. Some studies use different ages as a cutoff, ranging from >70 years to >85 years [31–33]. In the present study, the patients were divided into three groups, according to the age (YO, MO, and OO) [24, 34], and take as starting point ≥75 years [35]. In literature, there are limited available data on RACS for the most elderly groups. In the present study, few significant differences were observed between groups regarding clinical data. Some studies argue that older age is related to fragility and lower physiological reserves [23]. In our study, only the OO group showed the lowest BMI (20.9 kg/m^2^). Normal/healthy BMI and ASA II were seen more frequently in all groups with cardiovascular and endocrine diseases being the most common comorbidities. According to our study, only cirrhosis was a risk factor 41.198 (adjusted HR, CI 95% 4.617–367.623) times higher than other comorbidities for OS, which is consistent with literature since these patients have less tolerance to oncology treatment [36]. Therefore, surgical approach and preoperative management should be tailored individually taking into account all these factors, rather than based only on the chronological age [22]. This leads us to report that minimally invasive approach could be a viable option in these patients. In particular, since robotics limits the impact of predisposing factors for complications development, such as avoiding large fluid shifts or hypothermia following a closed abdomen and reduces collateral damage and tissue trauma, it may result in improved healing and faster return to functional baseline [22, 23]. The majority of patients in this study had advanced tumor at preoperative staging (stage II or III), and after MDT evaluation a total of 23 patients (30.2%) underwent neoadjuvant therapy. Dodaro et al. reported that better outcomes of neoadjuvant therapy can be achieved when this treatment is associated with a correct surgical technique “total mesorectal excision” (TME) [37]. Since many trials demonstrated that neoadjuvant therapy is associated with a decrease in local recurrence rates and an increase in the OS in all patients regardless of age [38], previous studies have examined the relation between elderly patients and local advanced rectal cancer [39, 40], a subanalysis focusing in pTNM stage II and III was carried out to comprehend the role of this patients in terms of CSS and CRR at 5 years. We observed that there was no significant difference in CSS (*p* = 0.706) and CRR (*p* = 0.925) between stages, however the CSS at 5 year was higher in YO (80%), MO (100%), and OO (40%) with pTNM stage III. Our results could demonstrate a CSS benefit for elderly patients with pTNM stage III and rectal cancer who complete a full course of recommended therapy and have RACS.

The most frequent tumor location in the present series was the lower rectum. It is well known that the primary goal of surgical intervention for rectal cancer is to achieve an oncologic cure while preserving function. Therefore, dissection must be carried out with extreme caution to avoid damage and to perform a nerve-sparing procedure, with adherence to the TME principles. Recent developments in robotic technology enable overcoming these difficulties caused by complex pelvic anatomy [41]. The international consensus project for multidisciplinary management of elderly patients with rectal cancer from the SICG (Italian Society of Geriatric Surgery), SIFIPAC (Italian Society of Surgical Pathophysiology), SICE (Italian Society of Endoscopic Surgery and new technologies), and the WSES (World Society of Emergency Surgery) suggest that laparoscopic TME in elderly fit patients with rectal cancer after a careful evaluation of patient's medical history, performance status, and tumor characteristics is feasible, and the role of robotic surgery for colorectal cancer resection may be associated with potential benefits over laparoscopy in terms of conversion rate, intraoperative blood loss, and hospital stay in general adult populations. Therefore, robotic surgery can be feasible in elderly patients with rectal cancer [42, 43]. Although the ROLARR trial did not demonstrate a clear difference between the robotic and laparoscopic approach for rectal cancer [44], recent studies support advantages of robotic surgery for rectal cancers [45, 46]. Oldani et al. reported that the robotic approach is safe, feasible, and offers many systemic benefits also with high ASA score, in terms of postoperative morbidity, hospital stay, first diet intake, first flatus canalization, and oncological outcome [47]. A recent systematic review carried out by Gravriilidis et al. showed a significantly lower conversion rates to open surgery in the robotic TME cohort than in the laparoscopic TME cohort from a multicenter study in Europe [48]. Mean operative time (279 min, SD 80.93), docking time (10 min, SD 3.50), and EBL (186 ml, SD 234.8) of the present study were similar to other studies on elderly populations using robotics, additionally with no conversion to open approach. [49–51] The short-term outcomes in the 30-day postoperative period showed a C-D ≤ II in 97.3% of patients, with only 2.6% requiring surgical management, and a 0% mortality rate with a mean postoperative hospital stay of 14.25 days (SD 12.03). de'Angelis et al. reported similar results as ours, in their propensity score matched analysis on elderly patients, in terms of surgical data and postoperative complications between laparoscopic and RACS [52].

Mean lymph node harvest with RACS was 20.9 (SD 12.33), with the OO group having the highest yield rate (mean of 24.56), which is consistent with the literature [46, 49–51]. Clinical and pathological TNM stages were different between the groups (*p* = 0.001). Clinical TNM stages II and III were most frequent in all groups, after MDT evaluation and RACS the final pTNM stages were I and II, this confirm the idea of aggressive treatment in the elderly and very elderly population [8].

There are few studies focusing on long-term oncological outcomes in elderly patients receiving RACS. Horsey et al. reported from the U.S. National Cancer Database a five-year OS of 63.7% in elderly patients (mean 73.2 years) subjected to RACS for CRC, which did not differ from patients submitted to laparoscopic approach, despite the robotic approach conferring a significantly increased chance of adequate lymphadenectomy and negative circumferential resection margin compared to laparoscopic approach [53]. Pinar et al., reporting the results from the Danish National database, demonstrated comparable rates of disease-free survival, all-cause mortality, and recurrence-free survival when comparing robot-assisted surgery with conventional laparoscopy in elderly patients with CRC [54].

To the authors' knowledge, the present study has the largest cohort from a single medical center to analyze and compare RACS in elderly and very elderly patients. The long-term oncological outcomes including OS, CSS, and CRR differed between the groups, with the OO group showing the lowest five-year OS and CSS (both 0%), and the lowest CRR (5% in the first year after surgery) in comparison to YO (41%), and MO (17%). It is important to note that the patients included in our study were older (≥75 years) than others cohorts and presented advanced stages at the time of diagnosis (TNM stage III), not being able to compare with other studies in terms of survival. [49–51].

To better comprehend the OS, CSS, and recurrence in elderly patients, a multivariate analysis was performed, showing that age is a risk factor for tumor recurrence (adjusted HR 10.50, 95% CI 1.868–59.047, *p* = 0.008), pathological TNM is not a risk factor for CSS (adjusted HR 0.84, 95% CI 0.239–3.001, *p* = 0.796), and resected lymph node number is a protective factor for tumor recurrence (adjusted HR 0.946, 95% CI 0.897–0.998; *p* = 0.042), and CSS (adjusted HR 0.93, 95% CI 0.869–1.000, *p* = 0.050) in elderly patients. Several studies support the robotic approach to provide a better lymph node harvesting over the laparoscopic approach on CRC[49, 53], with the present study confirming that lymph node harvesting through robotic approach is a protective factor in elderly patients with a possible impact on tumor recurrence and CSS.

The current study has several limitations. First, although the database is prospectively maintained the study was performed retrospectively with possible selection bias. Second, this is a monoinstitutional study with data from a national tertiary referral center with high volume for CRC. This could lead to possibly high-quality results derived from a high clinical and surgical experience on CRC and RACS. This could limit the possible comparison with other series from other centers. Third, even if the present study has the largest cohort from a single medical center to analyze and compare RACS in elderly and very elderly patients, the cohort size was still limited to 76 patients which is still small in numbers. Also, the division into subgroups, with relatively small number of patients (YO, *n* = 48; MO, *n* = 19; OO, *n* = 9), might have affected the statistics. Fourth, although the patients were old, the majority had a low ASA score with a relatively low BMI. Fifth, this series included a highly homogeneous population of Korean ethnics, which may limit the results for other ethnicities. International multicentric studies should be performed to overcome this limitation. Sixth, selection bias and long-term outcomes were not compared between each stage.

## 5. Conclusion

RACS is safe, feasible, and well tolerated for elderly and very elderly patients. Elderly patients demonstrated postoperative clinical outcomes and complications comparable to similar studies in robotic surgery for this population. Age should not be considered a limiting factor for CRC surgery, including robot-assisted surgery. Preoperative selection and assessment are crucial. More studies, internationally based, with a wider series, should be performed to demonstrate the beneficial effects of robotic surgery in the elderly.

## Figures and Tables

**Table 1:** Clinical data in elderly patients with RACS.

Clinical characteristics	Age 75–80 (*n* = 48)	Age 81–85 (*n* = 19)	Age ≥86 (*n* = 9)	*p* value
*Sex*, *female*^*∗*^	30 (62.5%)	5 (26.3%)	5 (55.6%)	**0.028**
*Body mass index*, *kg/m*^*2*^^*∗∗*^	24.1 (3.69)	24.4 (3.07)	20.9 (2.86)	**0.030**
*Comorbidities* ^*∗*^				0.128
Endocrine	7 (14.6%)	3 (15.8%)	2 (22.3%)	
Cardiovascular	23 (47.8%)	9 (47.4%)	3 (33.3%)	
Respiratory	1 (2.1%)	2 (10.5%)	0 (0.0%)	
Cirrhosis	7 (14.6%)	2 (10.5%)	0 (0.0%)	
Previous cancer	3 (6.3%)	3 (15.8%)	0 (0.0%)	
No comorbidities	7 (14.6%)	0 (0.0%)	4 (44.4%)	
*Carcinoembryonic antigen, ng/ml* ^*∗∗*^	3.91 (4.25)^a^	4.65 (4.87)^a^	10.30 (11.39)^b^	**0.010**
*ASA* ^*∗*^				0.645
I	2 (4.2%)	0 (0.0%)	0 (0.0%)	
II	39 (81.3%)	16 (84.2%)	6 (66.7%)	
III	7 (14.5%)	3 (15.8%)	3 (33.3%)	
*Radiological TNM* ^*∗*^				0.129
Stage I	3 (6.3%)	0 (0.0%)	1 (11.1%)	
Stage II	11 (22.9%)	1 (5.3%)	0 (0.0%)	
Stage III	31 (64.6%)	15 (78.9%)	6 (66.7%)	
Stage IV	3 (6.3%)	3 (15.8%)	2 (22.2%)	
*Neoadjuvant treatment, yes* ^*∗*^	14 (60.8%)	8 (34.7%)	1 (4.3%)	0.127
*Type of neoadjuvant treatment* ^*∗*^				0.056
CT	0 (0.0%)	1 (5.3%)	1 (11.1%)	
SCRT	2 (4.2%)	0 (0.0%)	0 (0.0%)	
LCCRT	12 (25.0%)	7 (36.8%)	0 (0.0%)	
*Adjuvant treatment, yes* ^*∗*^	14 (29.2%)	4 (21.1%)	(0.0%)	0.173

^*∗*^ *n* (%); ^*∗∗*^mean (SD). SD, standard deviation; CT, chemotherapy; SCRT, short-course radiotherapy; LCCRT, long-course preoperative chemoradiotherapy. Significant *p* values are in bold.

**Table 2:** Surgical and postoperative outcomes of elderly patients submitted to RACS: surgical data.

Surgical data	Age 75–80 (*n* = 48)	Age 81–85 (*n* = 19)	Age ≥86 (*n* = 9)	*p* value
*Location* ^*∗*^				0.839
Colon	2 (4.2%)	2 (10.5%)	0 (0.0%)	
Upper rectum (≥10 cm from AV)	6 (12.4%)	2 (10.5%)	1 (11.2%)	
Mid rectum (>5 and ≤ 10 cm from AV)	13 (27.1%)	4 (21.1%)	4 (44.4%)	
Lower rectum (≤5 cm from AV)	27 (56.3%)	11 (57.9%)	4 (44.4%)	
*Procedure* ^*∗*^				0.147
RH	1 (2.1%)	2 (10.5%)	0 (0.0%)	
AR	3 (6.3%)	0 (0.0%)	0 (0.0%)	
LAR	19 (39.6%)	8 (42.1%)	5 (55.6%)	
uLAR + DS	12 (25.0%)	1 (5.3%)	3 (33.3%)	
ISR + CAA	10 (20.8%)	5 (26.3%)	0 (0.0%)	
APR	3 (6.3%)	2 (10.5%)	0 (0.0%)	
Hartmann in PE	0 (0.0%)	1 (5.3%)	1 (11.1%)	
*Operation time, min* ^*∗∗*^	280.15 (84.04)	290 (78.88)	253 (69.91)	0.538
*Estimated blood loss, ml* ^*∗∗*^	133.3 (234.8)	290.0 (17.88)	253.3 (69.9)	**0.009**
*Clavien-Dindo classification* ^*∗*^				1.00
≤II	46 (95.8%)	19 (100.0%)	9 (100.0%)	
≥III	2 (4.1%)	0 (0.0%)	0 (0.0%)	
*Complications* ^*∗*^				0.144
Anastomotic leakage	2 (4.2%)	2 (10.5%)	0 (0.0%)	
Intraabdominal abscess	1 (2.1%)	3 (15.8%)	0 (0.0%)	
Wound infection	1 (2.1%)	0 (0.0%)	1 (11.1%)	
Ileus	7 (14.6%)	5 (26.3%)	2 (22.2%)	
Bleeding	1 (2.1%)	0 (0.0%)	0 (0.0%)	
Pulmonary	1 (2.1%)	0 (0.0%)	1 (11.1%)	
No complication	35 (72.8%)	9 (47.4%)	5 (55.6%)	
*Re-operation, yes* ^*∗*^	2 (4.1%)	0 (0.0%)	0 (0.0%)	1.000
*Temporary ileostomy, yes* ^*∗*^	26 (54.2%)	13 (68.4%)	3 (33.3%)	0.232
*Permanent colostomy, yes* ^*∗*^	4 (8.3%)	2 (10.5%)	1 (11.1%)	1.000
*Postop. hospital stay, days* ^*∗∗*^	13.77 (14.03)	13.58 (7.98)	18.22 (6.18)	0.579

^*∗*^ *n* (%); ^*∗∗*^mean (SD); AV, anal verge; SD, standard deviation; *p*RH: right hemicolectomy; AR, anterior resection; LAR, low anterior resection; uLAR, ultralow anterior resection; DS, diverting stoma; ISR, intersphincteric resection; CAA, colo-anal anastomosis; APR, abdominoperineal resection; PE, pelvis exenteration. Significant *p* values are in bold.

**Table 3:** Oncologic outcomes in elderly patients with RACS: pathological data.

Pathologic data	Age 75–80 (*n* = 48)	Age 81–85 (*n* = 19)	Age ≥86 (*n* = 9)	*p* value
*T stage* ^*∗*^				0.900
(y)pT1	2 (4.2%)	0 (0.0%)	1 (11.1%)	
(y)pT2	12 (25.0%)	1 (5.3%)	0 (0.0%)	
(y)pT3	31 (64.6%)	15 (78.9%)	6 (66.7%)	
(y)pT4	3 (6.3%)	3 (15.8%)	2 (22.2%)	
*N stage* ^*∗*^				0.184
(y)pN0	29 (60.4%)	15 (78.9%)	4 (44.4%)	
(y)pN1	12 (25%)	1 (5.3%)	2 (22.3%)	
(y)pN2	7 (14.6%)	2 (15.8%)	3 (33.3%)	
*Histology* ^*∗*^				1.000
Adenocarcinoma	46 (95.8%)	18 (94.7%)	9 (100.0%)	
Other	2 (4.2%)	1 (5.3%)	0 (0.0%)	
*Pathological TNM* ^*∗*^				0.127
(y)pStage I	17 (35.4%)	2 (10.5%)	2 (22.2%)	
(y)pStage II	13 (27.1%)	9 (47.4%)	1 (11.1%)	
(y)pStage III	15 (31.3%)	5 (36.3%)	5 (55.6%)	
(y)pStage IV	3 (6.3%)	3 (15.8%)	3 (11.1%)	
*Resected lymph nodes* ^*∗∗*^	20.60 (12.70)	20.16 (11.93)	24.56 (11.84)	0.648

^*∗*^ *n* (%); ^*∗∗*^mean (SD); SD, standard deviation.

**Table 4:** Analysis of risk factors associated with OS in elderly patients with CRC.

Characteristic	Cox proportional-hazards models			
	Unadjusted HR (95% CI)	*p* value	Adjusted HR (95% CI)	*p* value
*Age groups*				
75–80 years	Reference		Reference	
81–85 years	2.026 (0.797–5.151)	0.138	2.291 (0.778–6.742)	0.132
≥86 years	6.779 (2.208–20.816)	**0.001**	25.553 (5.600–116.596)	**0.001**
*Comorbidities*				
No comorbidities	Reference		Reference	
Endocrine	2.354 (0.381–14.550)	0.357	5.808 (0.832–40.563)	0.076
Cardiovascular	1.575 (0.349–7.116)	0.555	4.952 (0.810–30.261)	0.083
Respiratory	2.099 (0.187–23.599)	0.548	5.331 (0.334–85.111)	0.236
Cirrhosis	6.592 (1.152–37.717)	**0.034**	41.198 (4.617–367.623)	**0.001**
Previous cancer	2.646 (0.362–19.322)	0.337	9.240 (0.886–96.362)	0.063

HR, hazard ratio; CI, confidence interval. Significant *p* values are in bold.

**Table 5:** Analysis of risk factors associated with CSS in elderly patients with CRC.

Characteristic	Cox proportional-hazards models			
	Unadjusted HR (95% CI)	*p* value	Adjusted HR (95% CI)	*p* value
*Age groups*				
75–80 years	Reference		Reference	
75–81 years	1.666 (0.468–5.929)	0.431	2.100 (0.522–8.454)	0.296
≥86 years	5.434 (1.290–22.895)	**0.021**	9.087 (1.734–47.611)	**0.009**
*Pathological TNM*				
(y)pStage I	Reference		Reference	
(y)pStage II	0.918 (0.081–10.415)	0.945	2.316 (0.149–36.025)	0.549
(y)pStage III	0.740 (0.091–5.994)	0.778	1.047 (0.108–10.190)	0.968
(y)pStage IV	1.091 (0.98–12.120)	0.944	0.824 (0.60–11.361)	0.885
*Resected lymph nodes*	0.943 (0.882–1.007)	0.081	0.928 (0.861–0.999)	**0.047**

HR, hazard ratio; CI, confidence interval. Significant *p* values are in bold.

**Table 6:** Analysis of risk factors associated with tumor recurrence in elderly patients with CRC.

Characteristic	Cox proportional-hazards models			
	Unadjusted HR (95% CI)	*p* value	Adjusted HR (95% CI)	*p* value
*Age groups*				
75–80 years	Reference		Reference	
81–86 years	2.133 (0.674–6.750)	0.198	2.726 (0.639–11.621)	0.175
≥86 years	4.535 (1.138–18.082)	**0.032**	8.368 (1.547–45.248)	**0.014**
*pT stage*				
(y)pT1	Reference		Reference	
(y)pT2	0.141 (0.009–2.282)	0.168	0.565 (0.027–11.925)	0.714
(y)pT3	0.464 (0.059–3.616)	0.463	1.248 (0.127–12.240)	0.849
(y)pT4	0.241 (0.015–3.893)	0.316	0.184 (0.008–4.094)	0.285
*N stage*				
(y)pN0	Reference		Reference	
(y)pN1	2.649 (0.808–8.685)	0.108	4.341 (1.127–16.717)	**0.033**
(y)pN2	3.033 (0.855–10.761)	0.086	6.669 (1.382–32.192)	**0.018**
*Resected lymph nodes*	0.968 (0.923–1.015)	0.182	0.932 (0.875–0.992)	**0.027**

HR, hazard ratio; CI, confidence interval. Significant *p* values are in bold.

## Data Availability

The data sets used to support the findings of this study are available from the corresponding author on reasonable request.
